# Performance improvement indicators of the Medical Records Department and Information Technology (IT) in hospitals

**DOI:** 10.12669/pjms.313.8005

**Published:** 2015

**Authors:** Sima Ajami, Saedeh Ketabi, Fatemeh Torabiyan

**Affiliations:** 1 Sima Ajami, Ph.D. Professor, Department of Health Information Technology, School of Medical Management and Information Sciences, Isfahan University of Medical Sciences, Hezarjerib Avenue, Isfahan, Iran; 2 Saedeh Ketabi, Ph.D. Associate Professor, Dept. of Management, School of Administrative Sciences and Economics, University of Isfahan, Isfahan, Iran; 3 Fatemeh Torabiyan, Master student of Health Information Technology, Isfahan University of Medical Sciences, Hezarjerib Avenue, Isfahan, Iran

**Keywords:** Information Technology, Indicator, Medical Records, Performance

## Abstract

Medical Record Department (MRD) has a vital role in making short and long term plans to improve health system services. The aim of this study was to describe performance improvement indicators of hospital MRD and information technology (IT).

**Collection of Data:**

A search was conducted in various databases, through related keywords in articles, books, and abstracts of conferences from 2001 to 2009. About 58 articles and books were available which were evaluated and finally 15 of them were selected based on their relevance to the study. MRD must be capable of supporting tasks such as patient care and continuity, institute management processes, medical education programs, medical research, communication between different wards of a hospital and administrative and medical staff. The use of IT in MRD can facilitate access to department, expedite communication within and outside department, reduce space with electronic medical records, reduce costs, accelerate activities such as coding by use of coding guide software and facilitate retrieval of records that will ultimately improve the performance of MRD.

## INTRODUCTION

Medical Records Departments (MRD) in the hospitals are supposed to have complete records of patient’s admission. Medical Records (MR) documentation in accordance with predetermined standards; medical information coding process; creation and maintenance of statistical information database for planning and budgeting for hospitals; organizing outpatient and emergency medical records.^1^,^2^ Some deficiencies in hospitals MR performance necessitate the ever increasing use of information technology (IT) Deficiencies such as poor performance on patients’ admission and issuance of compiled instructions; lack of appropriate standard archive; weakness in informing referrals to MRD; incomplete records tracking system; incomplete MR coding; lack of effective and efficient use of information and records retrieval just to mention a few. 3-4

The question arises that how IT can improve the performance of medical records? The aim of this manuscript was to describe performance improvement indicators of the MRD of hospitals and IT through unsystematic review.

## METHODS

This study was divided into three phases: literature collection, assessing, and selection. The study was conducted by searching in a number of available databases such as Direct, PubMed, Proquest, Springer, Google, and SID through keywords of information technology, medical records, performance improvement, performance assessment, and indicators in texts and abstracts of articles, books, and proceeding in conferences from 2001 to 2014. About 59 articles and books were found and evaluated of which finally 15 cases were selected based on their relevance to this study.

## RESULTS

The benefits and IT applications in health system can be referred to empowering employees, the exchanging possibility of information between health care institutions, medical ethics, efficiency and effectiveness, online education, communication between patients and doctors, increasing geographic range of health services and health, increasing the health services quality and also increasing access to judicial services. Information Technology in healthcare department has provided many facilities such as obtaining information, medical advice and remote health for human society.^5^ MRD is also referred to as the hospital information pulse, has high potential for greater use of IT.^1^ Today IT in MRD is not only a competitive advantage, but it is also seen as a competitive necessity. IT applications can affect the performance improvement of MRD; applications such as quick responding to clients and staff by use of IT and hospital information management system, reduction of clients waiting time, increasing MR maintenance quality by electronic or scanned Medical records, enhancing security and confidentiality of information using access levels for each user allowed to use the system, information sharing through network and internet between different parts and in higher levels between hospitals which will lead to knowledge sharing and help medical education and research advancement, reduction of the costs using paperless system and reducing the space occupied by the paper MR and many other applications. Unlike the benefits and advantages mentioned above, some cases can be noted that lead to lack of proper implementation of IT in hospitals; such as attitudinal and behavioral constraints of staff, lack of technical infrastructure and software commensurate with performance, lack of funds by administrators to implement electronic health records, lack of proper technical support, and lack of experts.^6^-^9^

### Characteristics of favorable indicators

Developed performance assessment indicators should possess characteristics of a SMART & D system (SMART & D: Specific, Measureable, Achievable, Realistic, Time Frame, and Database).^10^ Indicators need to be meaningful, up-to-date, evidence-based, and repeatable while they are able to support assessment. Performance key indicators lead to promotion of user systems accountability and provide opportunities to compare organizations. Increased awareness of quality and safety in healthcare shows the importance of performance and quality assessment even more.^11^

If documentation follows standard quality and quantity, Medical Records are reduced and patients’ health is guaranteed. This important objective is achieved through application of IT in processes and MR staff which lead to improved communication, increased productivity, developed and continuous information sharing between hospitals, health centers, doctors, and patients. To identify performance indicators of MRD, the indicators mentioned in different studies are mentioned in Table-I.

**Table-I:** Indicators of performance assessment of MRD in different studies.

Researcher (year)	Units or departments	Indicators of performance assessment
Ajami et al., 2012 12	Admission	A: human resources- experience, education, and social relations; B: equipment; C: admission site and space; D: client satisfaction; E: processes
	Archive	A: human resources, experience, education; B: site and space of archive unit; C: equipment; D: client satisfaction; E: contents of MR including structure and content; F: security measures such as confidentiality, crisis management, processes
	Coding	A: human resources; B: equipment; C: client satisfaction; D: processes
	Statistics	A: human resources including number of employees, experience, education; B: equipment; C: client satisfaction; D: processes
Ajami et al., 2010 13	Admission	A: amount of client satisfaction, number of clients, amount of other staff satisfaction from admission, amount of complaints from admission; B: average waiting time of clients, number of methods for giving information to clients; C: training per employee, ratio of admission approvals to overall approvals of committee of medical documents, ratio of implemented legislation to overall approvals of internal meetings; D: turnover index of beds, occupancy index of beds
	Coding	A: number of formal clients for research; B: average registered code, average time of coding, average time of index, rate of error in coding; C: time of training documentation to doctors, coding training for technician, number of books; D: allocated financial-administrative credit
	Archive	A: number of formal clients, average time of responding official clients, number of informal clients, average time of responding informal clients, amount if complaints from MRD; B: number of files with empty forms; existing deficiencies in MR, existing deficiencies in MR according to type of the deficiency, time needed for file recovery, rate of compliance with safety standards; C: professional training, MR training capita for medical staff, ratio of MR approvals to overall approvals of committee of medical documents, ration of implemented legislation to overall approvals of medical documents committee, amount of correct usage of terminal digit system; D. amount of Rial credit for MRD, deductions due to incomplete Medical records, time required for billing records, time interval between discharge ordering and settlement
	Statistics	A: Satisfaction of provincial statistic headquarters; B: average hours spent on internet usage, statistics training capita, average error reported in statistics reports; C: average hours spent on internet, training capita, percentage of statistic approvals to overall approvals of economic and statistics assessment committee.

As explained in Table-I, various methods and indicators have been used for assessment of MRD by different researchers. However, due to lack of developed indicators there is lack of standardization and proportional performance measures related to activities of MRD. In this study, performance assessment indicators have been selected with the help of previous studies (Table-II).

**Table-II:** MR Performance Assessment Indicators.

Critical performance indicator	Indicator	Numerator	Denominator
Learning and growth	Ratio of professional staff MR	Number of MR professional staff	Number of staff based on chart
	Average training courses	Training courses held	Number of courses needed
	Percentage of trained staff to all staff employed in MR	Number of trained staff	All MR staff
	Ratio of documentation principles workshops to all training courses	Number of workshops held on documentation principles	Overall number of courses held
	Percentage of staff who have passed general courses based on validation measures	Number of employees who have spent general courses	Overall number of staff
	MR committee meeting in accordance with guidelines	Number of sessions	Overall number of meetings based on guidelines
	Ratio of training curses held for users in order to use system and software programs	Number of sessions held to train how to use system and software	Overall number of training courses held
Process	Amount of file deficiencies MR information	Number of deficient medical MR in a specific time period	Overall number of MR discharged
	Amount of deficiency elimination	Number of MR whose deficiency was eliminated	Overall number of deficient MR in a specific time period
	Amount of file deficiencies based on documentation group	Number of file deficiencies based on documentation group	Overall number of MR discharged
	Accuracy of MR archive	Number of MR archived correctly	Overall number of MR archived
	Average coded MR in a specific time period	Number of MR coded in a specific time period	Number of patients discharged at the same time period
	Percentage of statistical reports provided in a specific time period	Number of statistical reports provided	At the same specific period
	Percentage of MR errors	Number of errors in MRD	To overall number of hospital errors
Quality of services	Average waiting time of patients for admission	Patients’ waiting time for admission	Overall number of patients for admission
	Percentage of clients in MRD	Number of clients	Overall number of hospitalized patients
	Average time for file recovery	Time spent for file recovery	Number of MR recovered
	Average time for MR filing	Time spent for filing	Number of filed MR
	Percentage of visits made to expected visits	Number of visits made	Overall expected visits
	Timing of admission for hospitalized patients	Time spent for admission	Overall number of hospitalized patients
Client satisfaction	Average time for answering the clients	Time taken for answering the clients	Overall number of clients
	Clients’ satisfaction from MRD	Score of checklist	Number of completed checklists
	Percentage of received complaints regarding performance of MRD	number of received complaints regarding performance of MRD	Overall number of hospital patients
Security and confidentiality	Security of information in case of a problem	Number of lost information	Overall information in HIS
	Software alarming in probable case of error in HIS	Number of alarms given in case of error in HIS	Overall alarms given by HIS
Costs	Ratio of archive space in the hospital to standard space	Amount of physical space for archive	Standard archive space
	Ratio of archive space to overall space of hospital	Amount of physical space for archive by meters	Overall space of hospital
	Costs of equipment and IT in MRD	Costs spent on equipment and IT in MRD	Overall costs allocated to MR

In this study, critical performance indicators of MRD were studied under seven main categories of learning and growth, process, service providing, client satisfaction, security and confidentiality, and costs along with their performance indicators based on each department and its responsibilities as well as calculation formula. Zhang has indicated client satisfaction as a principle for using IT based on high quality performance.^14^ Given above critical indicators, IT can be useful in improvement of performance assessment results.^15^

## CONCLUSIONS

Promotion of Medical Records indicators along with identification of developed performance indicators which include all activities of four units in MRD can affect quality of healthcare services. Employees need both technical and communicative skills in order to improve their performance. This goal can be only achieved through knowledge, expertise, and training. Financing hospitals or participation of private sector, proper technical support from information systems, and presence of experienced experts who are able to deal with software and hardware problems will be greatly effective in successful usage of information technology.
